# A mixed-ethnicity myoclonus-dystonia patient with a novel *SGCE* nonsense mutation: a case report

**DOI:** 10.1186/s12883-021-02530-z

**Published:** 2022-01-05

**Authors:** Meliza Angelica J. de Leon, Raymond L. Rosales, Christine Klein, Ana Westenberger

**Affiliations:** 1 Neuroscience Institute, Department of Neuroscience & Behavioral Medicine, University of Santo Tomas Hospital, España Boulevard, Sampaloc, 1015 Manila, Metro Manila Philippines; 2 Institute for Neurosciences, St. Luke’s Medical Center, Quezon City, Philippines; 3 Institute of Neurogenetics, University of Lübeck, Lübeck, Germany

**Keywords:** *Myoclonus*, *Dystonia*, DYT*-SGCE*, Case report

## Abstract

**Background:**

Myoclonus-dystonia is a rare movement disorder with an autosomal dominant inheritance pattern characterized by a combination of myoclonic jerks and dystonia that may have psychiatric manifestations. Our aim is to present neurologic and psychiatric phenotypic characteristics in the first Filipino bi-ethnic myoclonus-dystonia patient and her father.

**Case presentation:**

We investigated a Filipino myoclonus-dystonia patient with a positive family history. This 21-year-old woman of mixed Filipino-Greek ethnicity presented with involuntary jerking movements of her upper extremities, head, and trunk. Her symptoms affected her activities of daily living which led her to develop moderate depression, mild to moderate anxiety, and mild obsessive-compulsive disorder (OCD). Her 49-year-old Greek father suffered from adolescence-onset myoclonus-dystonia.

**Conclusion:**

Genetic testing revealed a novel epsilon-sarcoglycan (*SGCE*) gene nonsense mutation c.821C > A; p.Ser274* that confirmed our clinical diagnosis. For co-morbid anxiety, depression, and OCD, this patient was given duloxetine, in addition to clonazepam for the myoclonus and dystonia.

## Background

Myoclonus-dystonia is a rare autosomal dominant movement disorder characterized by a combination of myoclonic jerks and dystonia [1–7]. About half of the patients have been found to harbor pathogenic variants in the epsilon-sarcoglycan gene (*SGCE*), a transmembrane protein of unknown function [1–4]. The murine *SGCE* gene is primarily transcribed from the paternal allele and pedigree analysis of families with this condition demonstrated a marked difference in penetrance depending on whether the disease allele came from the mother or the father [8]. This likewise demonstrates an assumed maternal imprinting mechanism in humans that may be responsible for the reduced penetrance of the condition in those who inherited the mutated allele from their mother [8]. The population prevalence of *SGCE*-linked myoclonus-dystonia is still unknown but some documents have considered it to be 1 in 500,000 in Europe [3, 4, 7]. It usually presents in childhood with a mean age of onset of 10 years with symptoms occurring earlier in girls compared to boys [3, 4]. Among the two phenotypes, myoclonus is regarded as the most disabling feature [3]. Motor symptoms are said to improve with intake of alcohol and dependence becomes a concern in some cases [1–4]. Psychiatric manifestations may likewise be part of this phenotype including anxiety-related disorders and obsessive-compulsive disorders [3]. The myoclonus remains fairly stable in adulthood but progression, regarded as spreading to previously unaffected body parts or worsening the severity of the already affected body parts, was reported in some cases [3].

Our aim is to present neurologic and psychiatric phenotypic characteristics in the first Filipino bi-ethnic myoclonus-dystonia patient and her father harboring a novel *SGCE* nonsense mutation.

## Case presentation

### Patient 1 (index patient)

**Fig. 1:**
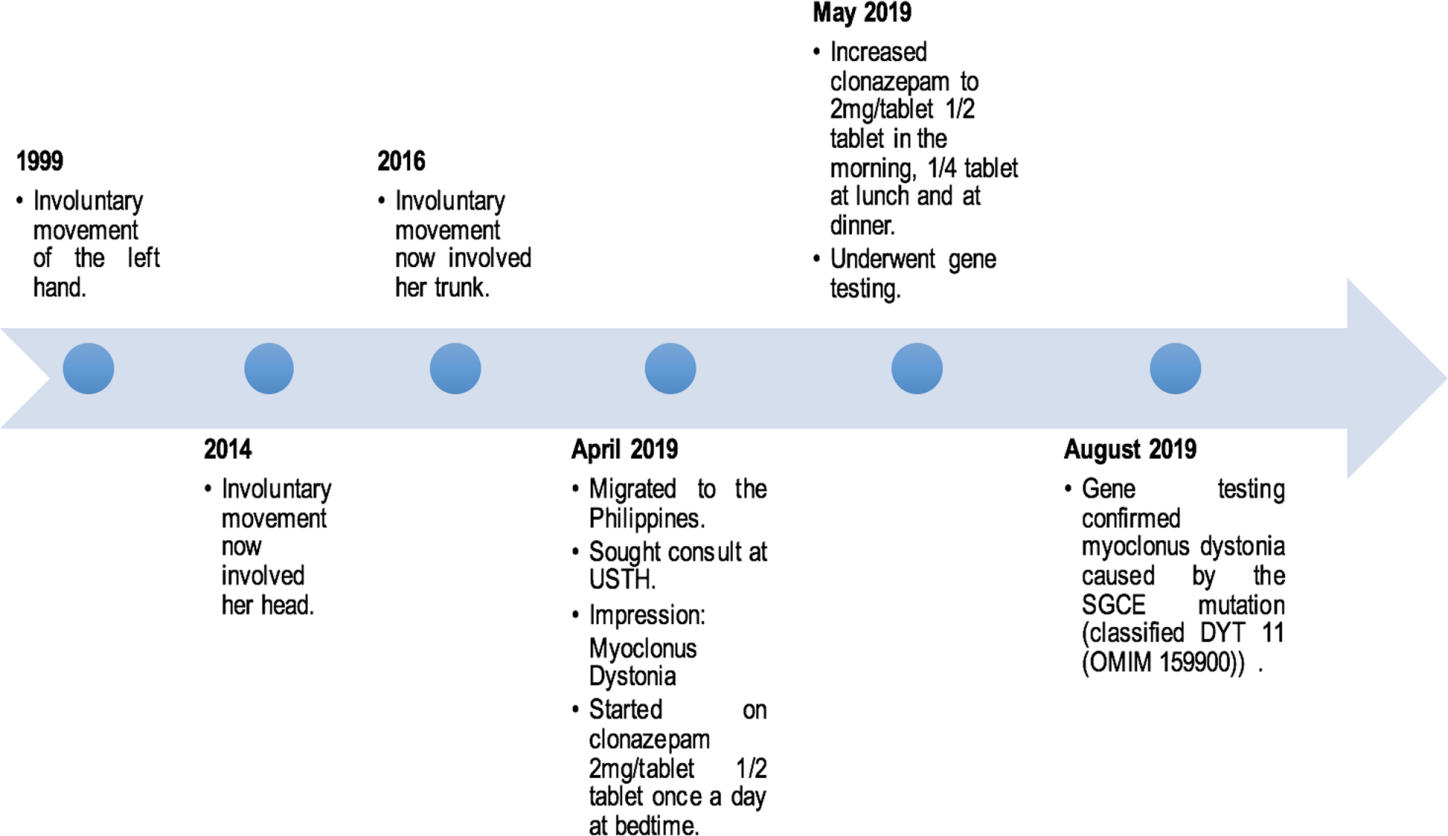
Timeline showing the symptom and treatment history from the onset until receiving a molecular diagnosis

Our index patient, a 21-year-old Filipino-Greek female college student, with right-hand dominance, consulted at our institution due to involuntary jerking movements of her upper extremities, head, and trunk. Her symptoms were first observed when she was 2 years old when her mother noted that she had instances where she would involuntarily throw her feeding bottle. Symptoms persisted and slowly progressed and at 15 years of age, the involuntary movements involved her head. When she was between 17 and 18 years of age, her trunk also became involved making it difficult for her to perform her activities of daily living, especially feeding. Due to this, she developed a depressed mood, lack of interest in her usual activities, episodes of insomnia, and weight gain. An unrecalled surgical procedure was offered in the past to which the family did not consent. No medications were initiated until she was seen at our institution (Fig. 1).

**Fig. 2:**
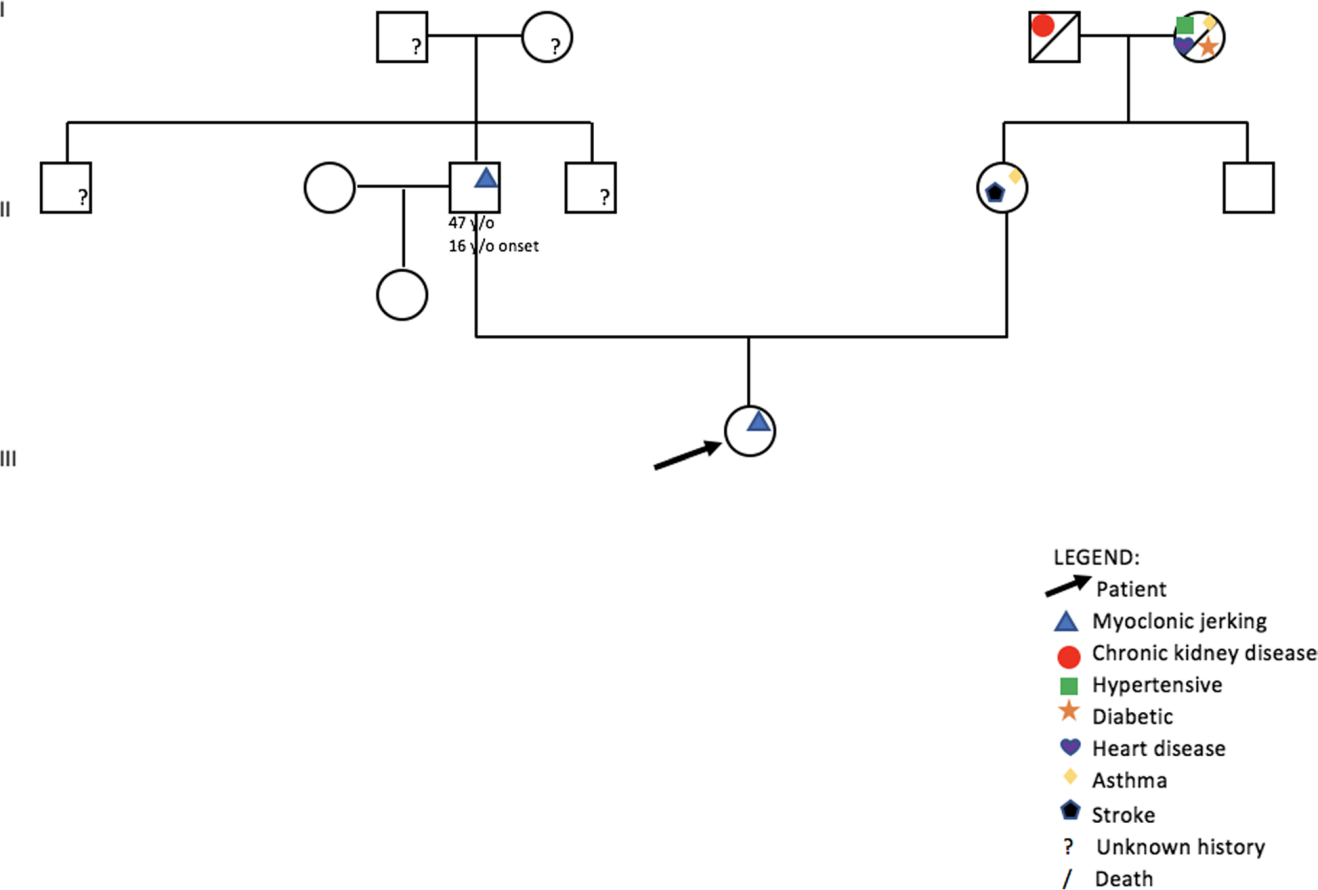
Pedigree of our proband (arrow)

Upon examination at the age of 21 years, she was noted to have weakness of both shoulder flexors, shoulder abductors, shoulder adductors, and notable abnormal involuntary myoclonic jerks on both upper extremities, head, and trunk. She likewise had focal dystonia on her left hand. The patient also complained of upper back pain and upon further assessment, was noted to have muscle spasms on her trapezius and rhomboids that we attributed to her dystonia. An x-ray was done that revealed dextroscoliosis. Her father (Patient 2) was also clinically diagnosed with myoclonus-dystonia. A distant paternal uncle likewise had an unknown movement disorder (Fig. 2). She has a 9-month-old sister who is currently asymptomatic.

Neuroimaging has been requested and performed at her previous consultations, was reviewed at our institution, and showed unremarkable results. A series of questionnaires were administered with the following scores: Unified Myoclonus Rating Scale (patient questionnaire: 9, myoclonus at rest: 18, stimulus sensitivity: 11, myoclonus with action: 13, functional test: 6, personal disability score: 2, global disability score: 2, negative myoclonus: 0), Unified Dystonia Rating Scale: 4.5, Fahn Marsden Scale: 8, Hamilton Anxiety Rating Scale: 22 (mild to moderate anxiety), Hamilton Depression Rating Scale 15: (moderate depression), Montgomery and Asberg Depression Rating Scale: 28 (moderate depression), and Yale-Brown Obsessive-Compulsive Scale (Y-BOCS) score: 3 (mild obsessive-compulsive disorder).

For co-morbid anxiety, depression, and OCD, this patient was given duloxetine, in addition to clonazepam for the myoclonus and dystonia. She was given orphenadrine citrate and paracetamol as needed which provided relief from her upper back pain.

Genetic testing involving Sanger sequencing of all *SGCE* exons and exon-intron boundaries revealed a nonsense mutation in exon 6 of the *SGCE* gene (c.821C > A; p.Ser274*) in the patient. This mutation has not been reported to date, neither in myoclonus-dystonia patients nor in over 150,000 individuals from the Genome Aggregation Database (gnomAD) [9].

### Patient 2 (father of the index patient)

A 49-year-old Greek man who was clinically diagnosed with alcohol-responsive myoclonus-dystonia with an age at onset of 16 years old. Upon examination, he was noted to have involuntary myoclonic jerks on his upper extremities, a postural tremor on his left upper extremity, cervical dystonia, and abnormal posturing of his hands when writing. He observed improvement of his myoclonic jerks and posturing with intake of alcohol. A series of administered questionnaires resulted in the following scores: Hamilton Anxiety Rating Scale: 3 (mild anxiety), Hamilton Depression Rating Scale: 3 (normal), Montgomery and Asberg Depression Rating Scale: 0 (normal) and Yale-Brown Obsessive Compulsive Scale: 0 (normal). The patient was given clonazepam for his myoclonic jerks and dystonia which improved his symptoms. Targeted Sanger sequencing of *SGCE* exon 6 in this patient revealed the same nonsense mutation (c.821C > A; p.Ser274*) as in his daughter (Patient 1).

## Discussion and conclusions

We have presented the case of a 21-year old, Filipino-Greek female with myoclonic jerks involving her upper extremities, head, and trunk with accompanying dystonia in her left hand, mild to moderate anxiety, moderate depression, and mild OCD. Upon review of her family history, it was noted that her Greek father and a distant paternal uncle likewise presented with similar symptoms, signifying a high likelihood of paternal transmission. Upon gene testing, it was revealed that she had myoclonus-dystonia caused by the *SGCE* mutation (classified DYT-*SGCE* (OMIM 159900)). She has a 9-month-old half-sister, likewise Filipino-Greek, who is currently asymptomatic. Counseling was provided for the patient and her family as well as close monitoring for symptoms as her younger sister may develop the aforementioned movement disorder in the near future. It is intriguing to consider whether a bi-ethnic origin of our patient would influence the transmission and the expression of the disease. The literature search revealed no genetic or functional study of a myoclonus-dystonia patient with a bi-ethnic origin. Nevertheless, given that the *SGCE* gene has been found to be maternally imprinted and thus expressed only from the paternal allele in numerous patients worldwide, there is no reason to assume that the Greek or Filipino ethnicity would influence this epigenetic phenomenon.

## Data Availability

The datasets used and/or analyzed during the current study is available from the corresponding author on reasonable request.

## Abbreviations

OCD: Obsessive Compulsive Disorder
*SGCE*: *Epsilon-Sarcoglycan* Gene
